# Early detection of neuropathy in leprosy: a comparison of five tests for field settings

**DOI:** 10.1186/s40249-017-0330-2

**Published:** 2017-09-01

**Authors:** Inge Wagenaar, Erik Post, Wim Brandsma, Dan Ziegler, Moshiur Rahman, Khorshed Alam, Jan Hendrik Richardus

**Affiliations:** 1 Department of Public Health, Erasmus MC, Rotterdam, The Netherlands; 2 KIT Health, Royal Tropical Institute, Amsterdam, The Netherlands; 3 Independent leprosy consultant, Amsterdam, The Netherlands; 4 Institute for Clinical Diabetology, German Diabetes Center at Heinrich Heine University, Leibniz Center for Diabetes Research, Düsseldorf, Germany; 5 Department of Endocrinology and Diabetology, Medical Faculty, Heinrich Heine University, Düsseldorf, Germany; 6 Rural Health Program, The Leprosy Mission International- Bangladesh, Nilphamari, Bangladesh

**Keywords:** Leprosy, Neuropathy, Detection, Subclinical, Field use

## Abstract

**Background:**

Early detection and treatment of neuropathy in leprosy is important to prevent disabilities. A recent study showed that the Nerve Conduction Studies (NCS) and Warm Detection Thresholds (WDT) tests can detect leprosy neuropathy the earliest. These two tests are not practical under field conditions, however, because they require climate-controlled rooms and highly trained staff and are expensive. We assessed the usefulness of alternative test methods and their sensitivity and specificity to detect neuropathy at an early stage.

**Methods:**

Through a literature search we identified five alternative devices that appeared user-friendly, more affordable, portable and/or battery-operated: the Neuropad®, Vibratip™, NC-Stat®DPNCheck™, NeuroQuick and the Thermal Sensibility Tester (TST), assessing respectively sweat function, vibration sensation, nerve conduction, cold sensation and warm sensation. In leprosy patients in Bangladesh, the posterior tibial and sural nerves that tested normal for the monofilament test and voluntary muscle test were assessed with the NCS and WDT as reference standard tests. The alternative devices were then tested on 94 nerves with abnormal WDT and/or NCS results and on 94 unaffected nerves. Sensitivity and specificity were the main outcomes.

**Results:**

The NeuroQuick and the TST showed very good sensitivity and specificity. On the sural nerve, the NeuroQuick had both a sensitivity and a specificity of 86%. The TST had a sensitivity of 83% and a specificity of 82%. Both the NC-Stat®DPNCheck™ and Vibratip™ had a high specificity (88% and 100%), but a low sensitivity (16% and 0%). On the posterior tibial nerve, the NeuroQuick and the TST also showed good sensitivity, but the sensitivity was lower than for the sural nerve. The Neuropad® had a sensitivity of 56% and a specificity of 61%.

**Conclusions:**

The NeuroQuick and TST are good candidates for further field-testing for reliability and reproducibility. The feasibility of production on a larger scale should be examined.

**Electronic supplementary material:**

The online version of this article (doi:10.1186/s40249-017-0330-2) contains supplementary material, which is available to authorized users.

## Multilingual abstracts

Please see Additional file 1 for translations of the abstract into the five official working languages of the United Nations.

## Background

Leprosy is a major cause of peripheral neuropathy in low resource countries, affecting sensory, motor and autonomic nerve functions. Complications of neuropathy are sensory loss and muscle weakness. Earlier studies found that 10% to 55% of newly diagnosed patients already showed one or both of these clinical symptoms [1–4], and that additionally, up to one fourth developed neuropathy during or after treatment [5]. Disabilities may develop if neuropathy remains untreated or is treated too late. Worldwide over three million people are living with disabilities due to leprosy [6]. Timely detection and treatment of neuropathy is essential to prevent disabilities.

Sensory nerve function impairment (NFI) is often the first symptom of leprosy neuropathy. Sensory NFI is typically detected with monofilament tests (MFT) or ballpoint tests, and motor function is assessed with voluntary muscle tests (VMT). It is assumed that when sensory impairment is clinically detectable, quite some damage has already been done to the nerves, the so-called subclinical neuropathy [7]. Methods to detect neuropathy in such early stages were studied in the INFIR (ILEP Nerve Function Impairment and Reactions) study [4]. To find the most sensitive methods, changes in the nerves of leprosy patients were monitored over time with multiple methods assessing different modalities of neuropathy. Nerve conduction studies (NCS) were found to be affected most frequently, followed by warm detection thresholds (WDT). These two methods were able to detect abnormalities in the nerves up to twelve weeks before MFT became abnormal. The NCS method assesses the large Aβ-fibres, responsible for perception of vibration, touch and pressure. The WDT method tests the small myelinated Aδ- and unmyelinated C-fibres, which mediate pain, warm- and cold sensation and govern the autonomic functions – e.g. sweating [8–11].

Even though NCS and WDT are highly valuable to detect early neuropathy, these devices are not optimal for field use in leprosy endemic areas. They are costly, require well-trained staff and need stable environmental (temperature) conditions and stable electricity supply. There is a need for cheap, easy-to-use, sensitive and reliable screening tools to detect early neuropathy. Our aim was to find potential alternative tests methods that can detect early neuropathy in leprosy patients and to compare the sensitivity and specificity of these methods when assessed in field conditions, using NCS or WDT as reference standard test.

## Methods

A literature search was conducted to identify user-friendly, portable devices used for the diagnosis of early neuropathy, regardless of the pathology. We covered the Embase, Medline and Cochrane databases and the search engine Google, using search terms related to ‘neuropathy’, ‘diagnosis’, ‘device’ and ‘simple’ (see Additional file 2).

The search resulted in 18 possibly eligible devices (Table 1). This selection was narrowed down based on the following requirements: costs less than €1500; availability; easiness of use – i.e. no extensive training required; and practicality and suitability for field conditions: small portable, battery-operated device providing direct results without the need for additional (computer) analyses. We also sought to cover different modalities of neuropathy.

**Table 1:** Results literature search and reasons for final selection

**Included tests**	**Modality**	**Reason selected**
NeuroQuick	Cold sensation	Portable, battery-operated
Neuropad	Sweat function	Easy, direct results, no training
NC-Stat DPNCheck	Nerve conduction	Portable, battery-operated
Vibratip	Vibration	Portable, battery-operated
Thermal Sensibility Tester	Warm sensation	Portable, battery-operated
**Excluded tests**	**Modality**	**Reason not selected**
NerveCheck	Warm, cold, pain and vibration sensation	Portable, battery operated, not available at the moment of this study
Tiptherm	Cold sensation	Weakest performance compared with other tests [19]
Neurometer	Nerve conduction	Expensive
Biothesiometer	Vibration	Expensive
Neurotip	Touch sensation	Low expected sensitivity, comparable to MFT
Neuropen	Pain sensation	Pain sensation loss is at a later stage than touch sensation loss [16, 40]
Sudoscan	Sweat function	Expensive
EzScan	Sweat function	Expensive
Bumps	Touch sensation	Low sensitivity, comparable to MFT
LDI-flare	Axon reflex–induced flare after heating skin (Doppler Imager)	Not portable, power needed
NervePace	Nerve conduction	Assesses motor latencies, while sensory conduction is more often affected in leprosy neuropathy
Neurosentinel	Nerve conduction	Similar to DPNCheck, only assessed Median nerve, which is less often affected in leprosy neuropathy
Thermotropic liquid Chrystal strip assessment	Skin temperature	Works when only one body side is affected (needs to be compared with normal side)

### Selected alternative devices

**Fig. 1:**
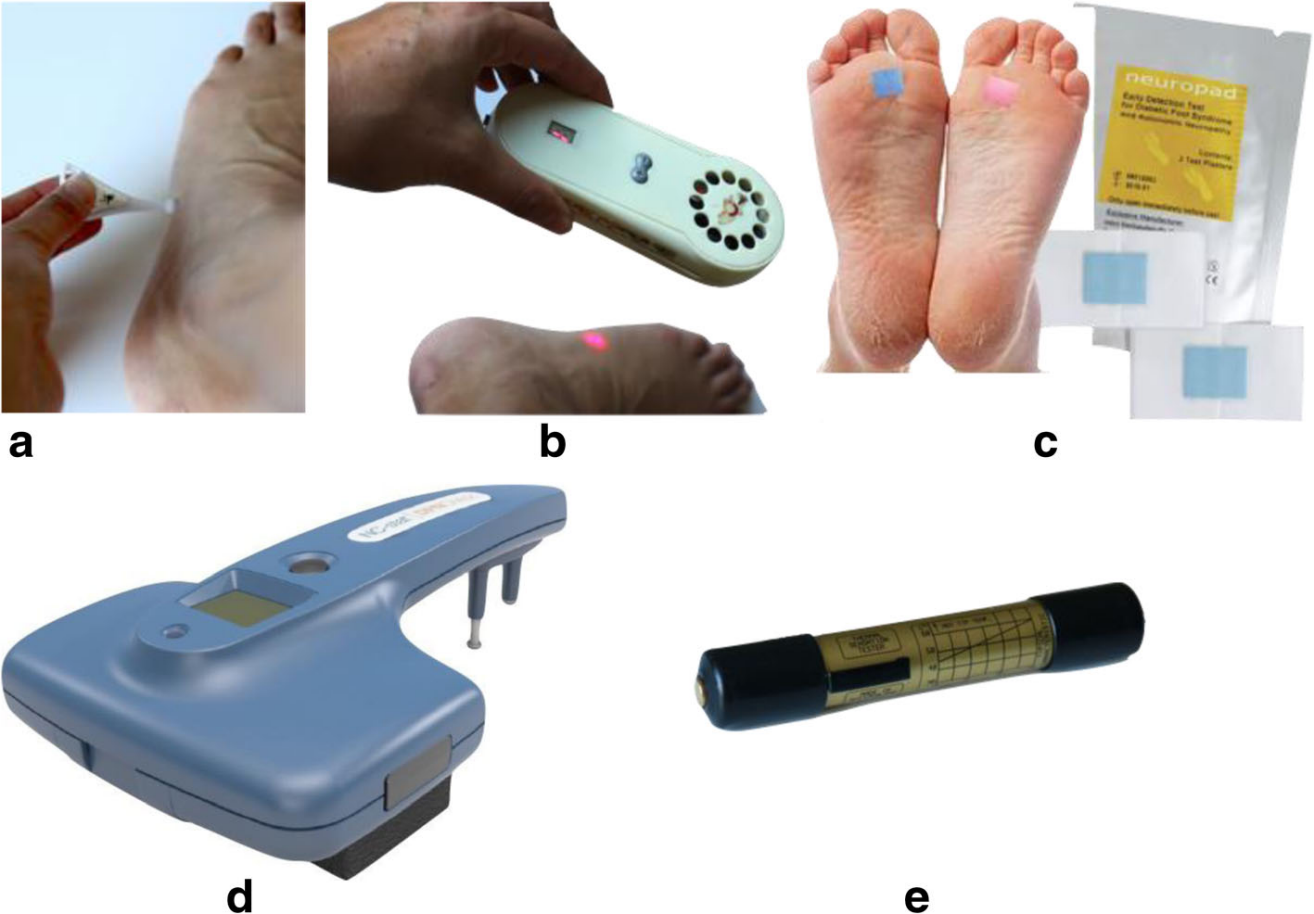
Pictures of the five devices. **a** Vibratip, **b** NeuroQuick, **c** Neuropad, **d** NC-stat DPNCheck, **e** Thermal Sensation Tester

The final selection consisted of the Neuropad® (TrigoCare International), Vibratip™ (McCallan Medical Limited), NC-Stat ®DPNCheck™ (NeuroMetrix, Inc.), NeuroQuick (Schweers) and the Thermal Sensibility Tester (TST) (World Health Organization). These are depicted in Fig. 1. For this study, the devices were kindly donated or lent to us by the producers. The TST was borrowed from the Netherlands Leprosy Relief organization.

The *Neuropad* is a patch designed to test the autonomic function of the diabetic foot by assessing sweat production. The colour of the patch changes from blue to pink due to a chemical reaction between the complex salt anhydrous cobalt-II-chloride and sweat on the skin. The main outcome is whether the patch turned completely pink after ten minutes, and the total time to complete colour change is recorded as well. The Neuropad is applied on the great toe or on the plantar surface of the foot between the first and second metatarsal head. The *Vibratip* is a pocket-sized device to test vibration sensation. This disposable, battery-operated device produces a stimulus of 128 Hz, comparable with a tuning fork, and is activated by pinching. The main outcome is whether vibration is felt.

The automated *NC-Stat DPNCheck* evaluates sensory nerve conduction of the sural nerve. Offering an alternative to standard NCS, this device is hand-held, battery-operated, fast and user-friendly, as general health care providers can handle it with minimal training. The sural nerve is stimulated with a 100 mA current, and the signal is detected orthodromically by a biosensor at 92.2 mm from the simulation probes. Conduction velocity and amplitude are the main outcomes.

The portable *NeuroQuick* device tests cold sensation with an airflow produced by the integrated adjustable fan. The fan speed is increased until the patient perceives the airflow. The outcome, fan speed on a 0 – 9 scale, is compared with a normal threshold to define whether the sensory nerve function is impaired.

The pen-sized *Thermal Sensibility Tester* (TST) assesses warm sensation. It was originally designed to test sensation in leprosy skin lesions [12]. Both ends of the pen have a little metal disc, one of which adjusts to room temperature and one becomes warm when switched on, with a temperature between 45 °C and 60 °C depending on environmental temperature [13]. The main outcome is whether the patient can distinguish the warm disc.

### Patients and controls

The subjects were all leprosy patients, diagnosed by enlarged peripheral nerves, skin lesions and/or a positive skin smear test. Patients who had normal MFT and VMT results for the posterior tibial and sural nerves were included when they had abnormalities in at least one sural or posterior tibial nerve with NCS and/or WDT test. Patients were excluded from the study when they suffered from other diseases that may affect the nerve function, e.g. diabetes. Patients under the age of 18 or over 70 years were excluded as well. Patients on prednisolone treatment were excluded from Neuropad testing, as a rare side effect of this drug is hyperhidrosis. To assess the specificity of the devices we also needed to test nerves with normal NCS and/or WDT. When one of the body sides of the included patients had normal nerve conduction and/or WDT test results for the sural or posterior tibial nerve, this nerve was taken as control. To achieve the calculated number of normal nerves we also had to include a number of leprosy patients with only normal NCS and/or WDT results as control subjects.

### Test procedure

The study took place in the Danish Bangladesh Leprosy Mission hospital at the Rural Health Program (RHP) department in Nilphamari, which is run by The Leprosy Mission International Bangladesh. Leprosy affected persons with normal VMT and MFT results for sural and posterior tibial nerves were sent to Nilphamari RHP from the nearby field clinics. There, they were again assessed again with these tests, according to a previous described protocol [14]. In short, sensory function was assessed on three test-sites for each nerve with a standard set of Semmes-Weinstein monofilaments, of which the 2 g filament represented the normal threshold for the foot. Motor function was assessed with the 0 – 5 Medical Research Council scale [15]. An MFT score under 3 and a VMT score of 5 were considered normal, as these are the thresholds used in large, recent leprosy studies [4, 16].

#### Reference standard tests

When MFT and VMT results were normal, the reference standard tests (sensory NCS and WDT tests) were carried out. Sensory NCS tests were performed for the sural nerve only, as we considered this method unreliable for the posterior tibial nerve. Sural NCS were recorded antidromically at 14 cm from the standard stimulation site. The test was done with the Neurocare 2000 W EMG machine (BioTech Ltd., Mumbai), and a nerve was considered affected when either amplitude or velocity was impaired. The WDT test was carried out using TSA II (MEDOC, Israel) for both sural and posterior tibial nerves, on respectively the mid-lateral border of the foot and the plantar aspect of the great toe. Both reference standard tests were done bilaterally, in an air-conditioned room by two experienced assessors who showed good inter-tester reliability (intraclass correlation coefficient of sural SNC, Velocity: 0.89; Amplitude: 0.99). The outcomes were compared with age-adjusted normal values previously established for the TENLEP trial at Nilphamari Rural Health Program (using the 97.5th percentile) [14]. However, for the posterior tibial nerve, WDT cut-off levels were set at the maximum temperature reached with the TSA, i.e. 50 °C. Applying this cut-off, none of the patients had abnormal values. Therefore, we redefined the threshold for abnormality by using the 95th percentile from the normative studies in the TENLEP trial, instead of the originally used 97.5th percentile.

#### Alternative devices

All five tests but one were performed for the sural nerve, which is the most commonly affected nerve in leprosy [17, 18]. The one exception was the Neuropad test, which was carried out on the posterior tibial nerve, since this test has been validated for the sole of the foot only. We tested the other two small nerve fibre tests – the NeuroQuick and the TST – on the posterior tibial nerve as well (Table 2). Not all patients were tested with all devices, for two reasons. First, only the nerve(s) identified as abnormal by one of the reference standard tests and their contralateral normal side were assessed with the alternative devices. Second, only the alternative devices that assessed the same type of nerve fibre – small (WDT) or large (NCS) – as the abnormal reference standard test were applied (see Table 2). For example, in a patient with abnormal sensory NCS result for one sural nerve we tested both sural nerves with NC-Stat DPNCheck and Vibratip (large fibre). The tests with the alternative devices were carried out on the same day as the reference standard tests, by one trained assessor (MR) who was blinded to the NCS and WDT results. The tests were done in the open air, without fan or air-conditioning to mimic field circumstances, in a random order following a randomization table created in Excel. Before testing, the assessor thoroughly explained and demonstrated the test procedures to the patient.

**Table 2:** Modality, nerve type and reference standard test for the alternative devices

Test	Modality	Nerve	Nerve fibre type	Reference standard test
NC-Stat DPNCheck	Conduction	Sural	Large fibre (Aβ)	NCS sural
Vibratip	Vibration	Sural	Large fibre (Aβ)	NCS sural
NeuroQuick	Cold sensation	Sural and posterior tibial	Small fibre (Aδ)	WDT sural/ PT
TST	Warm sensation	Sural and posterior tibial	Small fibre (C)	WDT sural/ PT
Neuropad	Sweat function	Posterior tibial	Small fibre (C)	WDT PT

*PT* posterior tibial nerve

The Vibratip, NeuroQuick and TST assessed the sural nerve at the mid-lateral border of the foot. With the Vibratip, the skin was touched twice for approximately one second, and randomly once with vibration and once without vibration. After the second touch, the patient was asked during which of the two touches vibration was felt. The test was repeated two more times. The patient’s vibration sensation was recorded as normal if the response was correct at least twice [19]. The outcome of the Vibratip tests was compared with the outcome of the NCS. A similar procedure was followed for the TST: the patient’s skin was touched twice for about three seconds, randomly once with the warmed disc and once with the ambient temperature disc. After the second touch, the patient was asked which of the two touches was perceived as warm. The test was repeated two more times. When the answer was correct at least twice, the patient’s warmth sensation was recorded as normal. The outcome of the TST was compared with the outcome of the WDT. The NeuroQuick test was started at fan speed level 0 and the device was then held for five seconds at a distance of 23 cm from the foot. This exact distance is shown by two crossing laser beams. When the patient did not perceive the airflow, the fan speed was increased by one level and held above the foot for another five seconds. This process was repeated until the airflow was perceived. The total procedure was repeated two more times and the mean of three measurements served as the outcome of the test. This outcome was classified as either normal or abnormal based on the cut-off levels of the normative test. The reference test used for comparison was WDT.

For the test with the NC-Stat DPNCheck, the standing patient placed the lower leg on a chair such that the muscles were relaxed [20]. The skin was cleaned with alcohol, a biosensor was inserted in the device and gel was applied on the stimulating probes. The sural nerves were stimulated for 10 – 15 s, just posterior to the lateral malleolus. Both velocity and amplitude, automatically corrected for skin temperature, were read directly from the device. The manufacturer suggests four severity categories: normal (velocity > 40 m/s and amplitude >4 μV), mild (velocity < 40 m/s, but amplitude >4 μV), moderate (amplitude 1 – 4 μV), and severe (amplitude <1 μV). The outcome of the DPNCheck was compared with the outcomes of the NCS. The Neuropad, NeuroQuick and TST were applied on the plantar side of the great toe. Similar procedures as described above were followed for NeuroQuick and TST. The Neuropad was applied after the patient had acclimatised for ten minutes with bare feet. For each Neuropad test, the total time to complete colour change from blue to pink was recorded as outcome, or if the change was not complete, the colour of the patch after ten minutes was noted (blue/pink or blue). The outcome of the Neuropad test was compared with the outcomes of the WDT. After all the tests had been finished, we asked the patient which of the five tests he or she valued the best and the worst and why. We also asked the assessor’s opinion on the practicality of the tests and we looked at the costs.

### Ethics

This is a sub-study of the TENLEP trial, for which ethical approval was given by the Bangladesh Medical Research Council (BMRC/NREC/2010-2013/533). An informed written consent was obtained from all participants.

### Analyses

Sample size calculations for an expected sensitivity of 0.60 and a confidence interval width of 0.20 resulted in 92 nerves per test. A similar number of normal nerves should be tested to determine the specificity at 0.60.

A normative study was carried out for the NeuroQuick to define the cut-off for abnormality. We tested the posterior tibial and sural nerves of 50 healthy males and 50 healthy females between 18 and 60 years of age. The NeuroQuick test procedure was as described above. As the outcomes for left and right body side did not differ significantly, the average of both body sides was used to calculate the normal thresholds. The correlation of NeuroQuick outcome with sex and age was tested with Spearman’s rho. Only age was significantly related with the NeuroQuick outcome for both nerves (sural: *r* = 0.64, *P* < 0.001; posterior tibial: *r* = 0.57, *P* < 0.001). We therefore used a regression equation to calculate an age-related cut-off for abnormality. For the sural nerve, the NeuroQuick result was considered abnormal when larger than 0.02 × Age + 3.4, and for the posterior tibial nerve when larger than 0.01 × Age + 3.3.

As primary outcome for our study, the sensitivity and specificity of each alternative device were calculated against the appropriate reference standard test – NCS or WDT– with their 95% confidence intervals. In addition, the positive and negative likelihood ratios and area under the curve (AUC) were calculated for each alternative device. A *P*-value <0.05 was considered to be statistically significant.

## Results

We enrolled 209 patients, and examined a total of 95 abnormal and 89 normal sural nerves with NCS, 94 abnormal and 90 normal sural nerves with WDT, and 75 abnormal and 115 normal posterior tibial nerves with WDT. The majority of patients had paucibacillary leprosy (67%) and were still on anti-leprosy multi-drug treatment (55%). On average leprosy had been diagnosed 11 months earlier. One patient was excluded because he was diagnosed with diabetes. The patients’ demographic and clinical characteristics are presented in Table 3.

**Table 3:** General characteristics of the patients (*n* = 209)

Characteristics			*SD*
Sex (% male)		57%	
Age (mean, years)		35.3	11.2
Height (mean, cm)		157	8
Weight (mean, kg)		51	9
Time since diagnosis (mean, months)		11	9
RFT (%)		46%	
RJ Classification (%)	TT	2%	
	BT	89%	
	BB	1%	
	BL	4%	
	LL	4%	
	PN	1%	
WHO classification (% PB)		67%	

*SD* standard deviation, *RFT* Released from anti-leprosy treatment, *RJ* Ridley-Jopling, *WHO* World Health Organisation

All patients tested with the Vibratip were able to detect the vibration for all three touches, irrespective of whether it was tested on an abnormal or normal nerve. TST and NeuroQuick outcomes are given in Table 4. Abnormal nerve conduction was indicated by the NC-Stat DPNCheck in 14% of the sural nerves. One nerve was mildly affected, 18 moderately affected and seven severely affected. The colour of the Neuropad had changed completely after ten minutes in 90 cases, and in 77 cases some blue was still visible. None of the pads remained completely blue. Mean time until complete colour change was 7.5 (range 2 to 10) minutes. Excluding the cases without complete colour change, the mean time was 4.9 min.

**Table 4:** Outcomes of the index tests and reference standard test, sensitivity and specificity (95% CI)

		Reference standard test						
Alternative devices		Abnormal	Normal	Sensitivity	Specificity	AUC	Positive Likelihood ratio	Negative Likelihood ratio
*Sural NCS (n)*		*95*	*89*					
NC-Stat	Abnormal	15	11	16%	88%	0.52	1.3	1.0
DPNCheck	Normal	80	78	(9 – 25)	(79 – 94)	(0.43 – 0.60)	(0.6 – 2.6)	(0.9 – 1.1)
Vibratip	Abnormal	0	0	0%	100%	0.50		1.0
	Normal	95	89	(0 – 4)	(96 – 100)	(0.41 – 0.58)	–	(1.0 – 1.0)
*Sural WDT (n)*		*94*	*90*					
TST	Abnormal	78	16	83%	82%	0.83	4.7	0.2
	Normal	16	74	(74 – 90)	(73 – 89)	(0.76 – 0.89)	(3.0 – 7.4)	(0.1 – 0.3)
NeuroQuick	Abnormal	81	13	86%	86%	0.86	6.0	0.2
	Normal	13	77	(78 – 92)	(77 – 92)	(0.80 – 0.92)	(3.6 – 10.0)	(0.1 – 0.3)
*Posterior tibial WDT (n)*		*75*	*115*					
Neuropad	Abnormal	42	35	56%	61%	0.59	1.5	0.7
	Normal	33	57	(44 – 67)	(51 – 72)	(0.51 – 0.67)	(1.1 – 2.1)	(0.5 – 1.0)
	Missing		23					
TST	Abnormal	60	50	83%	57%	0.70	1.9	0.3
	Normal	12	65	(72 – 91)	(47 – 66)	(0.63 – 0.76)	(1.5 – 2.4)	(0.2 – 0.5)
NeuroQuick	Abnormal	67	68	93%	41%	0.67	1.6	0.2
	Normal	5	47	(85 – 98)	(32 – 50)	(0.60 – 0.74)	(1.3 – 1.9)	(0.1 – 0.4)

*AUC* area under the curve, *NCS* Nerve Conduction Studies, *TST* Thermal Sensibility Tester, *WDT* Warm Detection Thresholds

The sensitivity, specificity, positive and negative likelihood ratios of all alternative devices are shown in Table 4. For the sural nerve, the NeuroQuick had the highest sensitivity and specificity (both 86%), closely followed by the TST (respectively 83% and 82%). The two devices assessing large fibre neuropathy in the sural nerves, the Vibratip and NC-Stat DPNCheck, had poorer outcomes. The sensitivity of the Vibratip was 0%; the specificity 100%. For the posterior tibial nerve, the NeuroQuick showed the highest sensitivity as well (93%), but its specificity was lower than that of the TST. The sensitivity and specificity of the Neuropad were average (56% and 60%). The highest positive likely hood ratio was that of the NeuroQuick for the sural nerve: 6.0 (3.6 – 10.0).

### Patients’ and assessor’s preferences

Sixty-eight patients were tested with at least four out of the five tests. More than half (53%) preferred the TST, mainly because they found it easy to feel a difference in warmth. The second favourite test was the Neuropad (25%), also because it was easy as it does not require any response from the patient. The Vibratip and the NeuroQuick were preferred by respectively 13% and 9%. When asked which test was least preferred, 54% answered the NC-Stat DPNCheck. The main reason was that it was too painful. The second least preferred test was the NeuroQuick (24%), since it was too difficult and third the Neuropad (22%), because it was seen as time consuming.

The assessor (MR) indicated he preferred the TST and Neuropad for their ease of use. He pointed out that for the patient the TST and the Vibratip are the best, since the warmth and vibration are easily detected. The assessor furthermore reported that the TST and Vibratip are the fastest tests.

### Costs

We have not performed a cost-effectiveness analysis, though still would like to present the costs of the devices. The retail price for Neuropads for clinics is $11 per test, containing two pads. The Vibratip costs $9. The list price for the NC-Stat DPNCheck is the highest of the alternative devices in our study, $1000 for the device and another $20 per sensor, which can be used to assess both left and right sural nerve in one patient. The TST and NeuroQuick are not available for purchasing, and therefore the prices are unknown. A paper published in 1989 describes that the cost of a TST was about $35 [13]. The TST and the NeuroQuick both work on two standard type AA batteries. Durability can be increased by using rechargeable batteries. The NC-stat DPNCheck uses a standard 3 V Lithium Ion battery. The Neuropads and the NC-stat DPNCheck biosensors are not suitable for recycling; these need to be disposed after using them for a single patient. The Vibratip can be used thousands of times over several months [21], but eventually needs to be disposed, as the battery is not replaceable.

## Discussion and conclusions

It is important to detect leprosy neuropathy at an early stage, as it can progress to nerve function impairment and subsequently may lead to disabilities. In the INFIR study, 20 – 50% of the newly diagnosed leprosy patients either had subclinical neuropathy at diagnosis or developed this during follow up [22]. Of the patients who had subclinical neuropathy, around 16% developed clinical NFI in the INFIR study. Preliminary data of the TENLEP trial show that clinical NFI developed in 8% of the subclinical patients. Even though treatment of subclinical neuropathy has not been successful [23, 24], knowing the subclinical status of a patient’s nerves allows to immediately start prednisolone treatment in the patients who do develop clinical NFI. In this study we compared user-friendly methods for detecting early leprosy neuropathy in field settings. We found that the NeuroQuick and TST are promising screening methods, especially for the sural nerve, since they show high sensitivity and specificity and are appreciated by both patients and assessor.

Both the NeuroQuick and the TST examine small fibre function. It must be noted, however, that the INFIR study found variation between the patients in the first affected modality and type of nerve fibre – large or small [25]. First of all, this indicates that the processes and patterns of neuropathy are different for each individual. Second, this has consequences for the assessment of early neuropathy. Adding a second test method that assesses large fibre function is recommended. Unfortunately, the two devices in our study testing large fibres, the NC-stat DPNCheck and the Vibratip, are not suitable for early detection of leprosy neuropathy. So far, NCS testing is the only reliable option to detect early large fibre neuropathy.

None of the alternative devices included in our study was new. The TST has been used to assess sensitivity of leprosy skin lesions [12]; the Neuropad, NeuroQuick, Vibratip and NC-stat DPNCheck have been used for diagnosis of diabetes neuropathy. Studies in diabetes patients showed generally good sensitivity and specificity for the detection of neuropathy [19, 26–31]. Although both small and large fibres are involved in diabetes neuropathy and in leprosy neuropathy, diabetes neuropathy is often symmetrical [32] and the order of affected modalities is somewhat different between the two diseases. In diabetes for example, cold perception proved the most sensitive thermal test [33], whereas in leprosy warm detection was more sensitive [34]. Furthermore, the tests in diabetes patients have been performed in a completely different setting and environment, namely western hospital settings. Therefore, it was necessary to test the devices in a leprosy endemic country with a subtropical climate, in a field setting with high temperatures and humidity. Interestingly, the TST, designed for testing in high environmental temperatures, showed good results. The temperature of the warm-end of the TST adapts to the ambient temperature, when between 15 °C and 45 °C. A graph with the correlation is depicted on the device.

The accuracy of the reference standard test, to which the new tests are compared, is an important point to consider. When the reference standard test is not 100% accurate, this has an effect on the sensitivity and specificity estimates [35]. NCS test results have been compared with nerve biopsies and were found to be accurate [36]. In addition, NCS testing is more reliable because it does not require a response from the subject and therefore objective. The WDT test, on the other hand, has never been compared with a gold standard in leprosy neuropathy. Furthermore, it requires a response from the subject and is therefore less objective. Perkins et al. describe that variances of 50% and higher can occur in thermal thresholds testing. However, since we use very high cut-off levels for normality (97.5%), we are confident that the sural nerves tested abnormal in our study are indeed abnormal. This is less the case for the WDT of the posterior tibial though. We could not rely on the upper thresholds for the posterior tibial nerve, since they mainly lay outside the measurement range of the TSA (50 °C). Therefore, we used a lower cut-off by taking the 95th percentile, but even with that method it was very difficult to include abnormal nerves.

Since in leprosy sensory function generally is affected before motor function, we did not include any assessments for motor nerves. Several other tests might be worthwhile to look at in future studies. We intended to include the NerveCheck (PhiMed Europe), which is a portable device that assesses four modalities: cold, warm, pain and vibration sensation [37]. Unfortunately, at the time of our study no NerveCheck device was available for field testing. Second, it might be interesting to look at skin wrinkling as an autonomic function test. Although this test takes long – 30 min – the results are quite promising in diabetes neuropathy [38, 39].

In conclusion, based on our results the Neuropad, NCstat DPNCheck and Vibratip do not qualify for further testing for detection of early leprosy neuropathy. For both the NeuroQuick and TST, however, we recommend to further study three different aspects. First, the options for further development and production on a larger scale should be examined. Second, repeatability and reproducibility should be determined for these two tests, and preferably assessed in different populations as well. Third, additional testing of the accuracy on the hands can give more information on the usability of the two tests in detecting early leprosy neuropathy in field settings, as upper extremity neuropathy is common as well.

## Additional files

Additional file 1: Multilingual abstract into the five official working langauges of the united Nations. (PDF 551 kb)

Additional file 2: Search terms literature study. Search terms used for the literature study. (DOCX 79 kb)

Additional file 3: Database ‘Early detection of neuropathy in leprosy’.sav. Clinical data of leprosy patients assessed for subclinical neuropathy, Nilphamari Bangladesh, 2015. (SAV 48 kb)

## Abbreviations

MFT: Monofilament test
NCS: Nerve Conduction Studies
NFI: Nerve Function Impairment
TST: Thermal Sensibility Tester
VMT: Voluntary Muscle Testing
WDT: Warm Detection Threshold
