# Supplementary material for: Early detection of neuropathy in leprosy: a comparison of five tests for field settings
Source: Infect Dis Poverty. 2017 Sep 1;6:115. doi: 10.1186/s40249-017-0330-2 (PMC5580225; doi:10.1186/s40249-017-0330-2)
Supplement: Supplementary file 1 — Multilingual abstract into the five official working langauges of the united Nations. (PDF 551 kb) [file 40249_2017_330_MOESM1_ESM.pdf]

Translation of the abstract into the five official working languages of the United Nations

**الاكتشاف المبكر لاعتلال الأعصاب في الجذام: مقارنة بين خمسة اختبارات لإعدادات الحقل**

إنجي فاخنار، إريك بوست، ويم براندسما، دان زيجلر، مشير الرحمن، خورشيد ألاماند جان هنريك ريكاردوس

**ملخص**

**خلفية** الكشف المبكر وعلاج الاعتلال العصبي في الجذام مهم للوقاية من الإعاقة. وأظهرت دراسة حديثة أن دراسات التوصيل العصبي (NCS) واختبارات حد الملاحظة الدافئ (WDT) يمكنها الكشف عن الاعتلال العصبي في مرض الجذام في وقت أقرب. مع ذلك هذان الاختباران ليسا عمليان في ظل الظروف الميدانية، لأنهما يتطلبان غرف تحكم في المناخ وموظفين مدربين تدريبا عاليا وتكاليف عالية. قمنا بتقييم جدوى طرق الاختبار البديل وحساسيتها ونوعيتها للكشف عن اعتلال الأعصاب في مرحلة مبكرة.
**الأساليب** من خلال البحث في التجارب السابقة حددنا خمسة أجهزة بديلة تبين سهولة استخدامها، ومعقولية سعرها، وكونها محمولة و / أو تعمل بواسطة بطارية وكانت: ®Neuropad، ™ Vibratip، ™ NC-Stat®DPNCheck، NeuroQuick وفاحص الإحساس الحراري (TST) وتم تقييم على التوالي وظيفة العرق، واستشعار الاهتزاز، والتوصيل العصبي، والاستشعار البارد والاستشعار الدافئ. في مرضى الجذام في بنجلاديش، تم تقييم الأعصاب الظنبوبية والربلية الخلفية التي اختبرت ووجدت طبيعية في اختبار الخيط البلاستيكي واختبار العضلات الإرادية مع دراسات التوصيل العصبي واختبارات حد الملاحظة الدافئ باعتبارها اختبارات قياسية مرجعية. ثم تم اختبار الأجهزة البديلة على 94 عصبا حيث كانت نتائج اختبارات حد الملاحظة الدافئ و / أو نتائج دراسات التوصيل العصبي غير طبيعية وكانت في 94 عصبا لم تتأثر. وكانت الحساسية والنوعية هي النتائج الرئيسية.
**النتائج** أظهر NeuroQuick وفاحص الإحساس الحراري حساسية ونوعية جيدة جدا. على العصب الربلي، كانت كل من حساسية ونَوْعِيَّة NeuroQuick 86٪. وكان حساسية فاحص الإحساس الحراري 83٪ والنَوْعِيَّة 82٪. وأظهر كل من NC-Stat®DPNCheck ™ وVibratip ™ نَوْعِيَّة عالية (88٪ و100٪)، ولكن حساسية منخفضة (16٪ و0٪). على العصب الظنبوبي الخلفي، أظهر NeuroQuick وفاحص الإحساس الحراري حساسية جيدة، ولكن أظهر حساسية أقل في العصب الربلي. كانت الحساسية في Neuropad® 56٪ والنَوْعِيَّة 61٪.
**استنتاجات** NeuroQuick وفاحص الإحساس الحراري يستحقان المزيد من الاختبار الميداني للموثوقية والتناتجية. وينبغي دراسة جدوى إنتاجهما على نطاق أوسع.

Translated from English version into Arabic through

TRANSLATORS
WITHOUT BORDERS

**麻风神经病变的早期诊断：应用于现场环境的 5 个测试的比较**

Inge Wagenaar, Erik Post, Wim Brandsma, Dan Ziegler, Moshiur Rahman, Khorshed Alamand Jan Hendrik Richardus

**摘要**

**引言：**麻风神经病变的早期诊断和治疗对于预防残疾是非常重要的。最近的研究显示神经传导功能检查（NCS）和暖检测阈值（WDT）测试能够在早期检测到麻风神经病变。然而，这两项检测不适用于现场环境，因为它们需要温控房间和训练有素的人员，并且费用较高。我们评估替代测试方法的有效性及其在早期阶段检测神经损害的敏感性和特异性。
**方法：**经过文献调研，我们共发现 5 种替代设备具有容易操作、费用能够承担，可移动或者可电池供电的特点， 这些设备是 Neuropad®， Vibratip™， NC-Stat®DPNCheck™， NeuroQuick 和 the Thermal Sensibility Tester (TST)，分别测定排汗功能，振动知觉，神经传导，冷知觉和暖知觉。在孟加拉国麻风病人中，经后胫骨和腓肠神经测试正常的人员采用 NCS and WDT 作为诊断参考对单丝和自愿肌肉测试。随后对 94 份 WDT and/or NCS 测试结果正常的神经和 94 份未受影响的神经进行测试，以敏感性和特异性作为主要结果。
**结果：**NeuroQuick 和 TST 显示出非常好的敏感性和特异性。NeuroQuick 在腓肠神经上的敏感性和特异性均为 86%。TST 的敏感性为 83%，特异性为 82%。NC-Stat®DPNCheck™和 Vibratip™的特异性较高（88%和 100%），但是敏感性很差（16%和 0%）。胫后神经测试中，NeuroQuick 和 TST 也表现出了较好的敏感性，但是低于在腓肠神经上的敏感性。Neuropad®敏感性为 56%，特异性为 61%。
**结论：**因为 NeuroQuick 和 TST 具有可靠性和可重复性，因此可以作为较好的候选测试手段。

应该审查其大规模生产的可行性。

Translated from English version into Chinese by Men-Bao Qian, through

CHINESE CENTER FOR DISEASE CONTROL AND PREVENTION
NATIONAL INSTITUTE OF PARASITIC DISEASES

**Détection précoce d'une neuropathie dans la lèpre: Une comparaison de cinq tests sur le terrain**

Inge Wagenaar, Erik Post, Wim Brandsma, Dan Ziegler, Moshiur Rahman, Khorshed Alamand Jan Hendrik Richardus

**Résumé**

**Contexte:** La détection et le traitement précoce d'une neuropathie dans la lèpre est importante pour éviter les handicaps. Une étude récente a montré que les Etudes de Conduction Nerveuse (ECN) et les tests de Seuils de Détection Chaude (SDC) peuvent détecter très tôt une neuropathie de la lèpre. Ces deux tests ne sont cependant pas pratiques sur le terrain, car ils nécessitent des pièces climatisées ainsi qu'un personnel hautement qualifié, et ils sont couteux. Nous avons évalué l'utilité de méthodes alternatives de test ainsi que leur sensibilité et spécificité pour détecter une neuropathie à un stade précoce.

**Méthodes** Par une recherche de documentation, nous avons déterminé cinq autres dispositifs faciles à utiliser, d'un prix plus abordable, portatifs et/ou fonctionnant sur batterie : le Neuropad®, le Vibratip™, le NC-Stat®DPNCheck™, le NeuroQuick et le Thermal Sensibility Tester (TST), évaluant respectivement la fonction de sudation, la sensation de vibration, la conduction nerveuse, la sensation de froid et la sensation de chaleur. Chez les patients lépreux du Bangladesh, les nerfs postérieurs tibial et sural que le test du mono-filament a trouvé normaux, et le dépistage volontaire du muscle, ont été évalués par les ECN et les SDC comme référence de dépistage normalisé. Les autres dispositifs ont été alors testés sur 94 nerfs avec des résultats anormaux des SDC et/ou ECN, et sur 94 nerfs non touchés. La sensibilité et la spécificité ont été les principaux résultats.

**Résultats** Le NeuroQuick et le TST ont montré une bonne sensibilité et une bonne spécificité. Sur le nerf sural, le NeuroQuick a eu une sensibilité et une spécificité de 86%. Le TST a eu une sensibilité de 83% et une spécificité de 82%. Le NC-Stat®DPNCheck™ et le Vibratip™ ont eu tous les deux une spécificité élevée (88% et 100%), mais une sensibilité basse (16% et 0%). Sur le nerf postérieur tibial, le NeuroQuick et le TST ont également montré une bonne sensibilité, mais la sensibilité a été plus basse que pour le nerf sural. Le Neuropad® a eu une sensibilité de 56% et une spécificité de 61%.

**Conclusions** Le NeuroQuick et le TST sont de bons candidats pour de futurs tests sur le terrain de fiabilité et de reproductibilité. La faisabilité d'une production à plus grande échelle devra être étudiée.

Translated from English version into French through

TRANSLATORS WITHOUT BORDERS

**Ранняя диагностика нейропатии в лепре: сравнение пяти тестов для полевых настройк**

Inge Wagenaar, Erik Post, Wim Brandsma, Dan Ziegler, Moshiur Rahman, Khorshed Alamand Jan Hendrik Richardus

**Реферат**

**Фон**: Раннее выявление и лечение нейропатии в лепре имеют важное значение для предотвращения инвалидности. Недавнее исследование показало, что исследования нервной проводимости (ИНП) и Warm Detection Thresholds (WDT) тесты могут обнаружить проказы невропатия в ближайшее время. Эти два теста не имеют практического применения в

полевых условиях, однако, поскольку они требуют климат-контрольных номеров и высококвалифицированных персоналов и высокой цены. Мы оценили полезность альтернативных методов тестирования и их чувствительность и специфичность для выявления нейропатии на ранней стадии.
**Методы**: С помощью поиска литературы мы выделили пять альтернативных устройств, которые появились удобные, более доступные, портативные и/или на батарейках: the проводимость, ощущение холода и теплое ощущение. В больных с лепрой в Бангладеш для моноволокно испытания и добровольного тестирования мышц оценивали с NCS и WDT в качестве эталонных стандартных тестов. Затем альтернативные устройства были протестированы на 94 нервы с аномальной продукцией WDT и/или результатами NCS и на 94 непораженных нервах. Чувствительность и специфичность были главными итогами.

**Результаты**: NeuroQuick и TST показали очень хорошую чувствительность и специфичность. ИкроножнЫЙ нерв, NeuroQuick имеют обе 86% чувствительности и специфичность. TST имеет 83% чувствительности и 82% специфичности. Оба NC-Stat®DPNCheck™ и Vibratip™ имеет высокую специфичность (88% и 100%), но низкую чувствительность (16% и 0%). На заднем большеберцовом нерве, NeuroQuick и TST также показали хорошую чувствительность, но чувствительность была ниже, чем на икроножном нерве. Neuropad® имеет 56% чувствительности и 61% специфичности.

Translated from English version into French by Hao-Qi Zhang

**Detección precoz de neuropatía leprosa: Comparación de cinco pruebas para ser utilizadas en el terreno**

Inge Wagenaar, Erik Post, Wim Brandsma, Dan Ziegler, Moshiur Rahman, Khorshed Alamand Jan Hendrik Richardus

**Resumen**
**Antecedentes**: La detección precoz y el tratamiento de la neuropatía leprosa es importante para prevenir discapacidades. Un estudio reciente reveló que las pruebas de estudios de conducción nerviosa (NCS) y de umbrales de detección de calor (WDT) pueden detectar la neuropatía leprosa más precozmente. Sin embargo, estas dos pruebas no son prácticas cuando se trabaja en el terreno debido a que requieren salas con control de temperatura y personal altamente especializado y son muy costosas. Evaluamos la utilidad de métodos de evaluación alternativos y su sensibilidad y especificidad para detectar neuropatías en una etapa temprana.
**Métodos**: Buscamos entre la literatura e identificamos cinco dispositivos alternativos que parecían fáciles de usar, más accesibles, portátiles y/o a pila: Neuropad®, Vibratip™, NC-Stat®DPNCheck™, NeuroQuick y Aparato de Sensibilidad Térmica (TST), y evaluamos la función de transpiración, la sensación de vibración, la conducción nerviosa, la sensación de frío y la sensación de calor respectivamente. En los pacientes con lepra en Bangladesh, los nervios tibial posterior y sural que resultaron normales para la prueba de monofilamento y la prueba del músculo voluntario fueron evaluados con NCS y WDT como pruebas estándar de referencia. Los aparatos alternativos fueron luego probados en 94 nervios con resultados WDT y/o NCS anormales y en 94 nervios no afectados. Los principales resultados fueron la sensibilidad y la especificidad.
**Resultados**: El NeuroQuick y el TST mostraron muy buena sensibilidad y especificidad. En el nervio sural, el NeuroQuick tenía sensibilidad y especificidad del 86%. El TST tenía una sensibilidad del 83% y especificidad del 82%. Tanto el NC-Stat®DPNCheck™ como el Vibratip™ tenían especificidad alta (88% y 100%), pero baja sensibilidad (16% y 0%). En el nervio tibial posterior, el NeuroQuick y el TST también mostraron buena sensibilidad, pero la sensibilidad era más baja que para el nervio sural. El Neuropad® tenía una sensibilidad del 56% y una especificidad del 61%.
**Conclusiones**: Los dispositivos NeuroQuick y TST son buenos candidatos para pruebas en el terreno debido a su confiabilidad y reproducibilidad. Debería examinarse la posibilidad de

producción a mayor escala.

Translated from English version into Spanish through

TRANSLATORS
WITHOUT BORDERS
