# Supplementary material for: Early detection of neuropathy in leprosy: a comparison of five tests for field settings
Source: Infect Dis Poverty. 2017 Sep 1;6:115. doi: 10.1186/s40249-017-0330-2 (PMC5580225; doi:10.1186/s40249-017-0330-2)
Supplement: Supplementary file 2 — Search terms literature study. Search terms used for the literature study. (DOCX 79 kb) [file 40249_2017_330_MOESM2_ESM.docx]

(neuropathy/exp OR 'neurologic disease'/de OR 'nervous system injury'/de OR (neuropath* OR polyneuropath* OR neuritis OR (nerv* NEXT/1 fibre*OR fiber*) OR ((nerv* OR neural OR nervous) NEAR/3 (degenerat* OR injur* OR lesion* OR disease* OR inflammat* OR damage* OR impair* OR necros* OR dysfunction* OR sign* OR symptom*)) OR neurodegenerat* OR neuroinflammat*):ab,ti) AND ((('diagnostic procedure'/de OR diagnosis/exp OR diagnosis:lnk OR examination/exp OR 'neurologic examination'/exp OR screening/exp OR 'sensory system examination'/de OR 'clinical assessment'/de OR 'functional assessment'/de OR 'medical assessment'/de OR (diagnos* OR examinat* OR screen* OR assess* OR monitor* OR analys* OR analyz* OR indicat* OR indentif*):ab,ti) AND (procedures/de OR equipment/exp OR devices/de OR 'medical device'/de OR (test* OR tool* OR procedure* OR equipment* OR instrument* OR technique* OR device* OR detect* OR ((diagnos* OR detect*) NEAR/3 (method* OR kit*))):ab,ti)) OR 'clinical assessment tool'/de OR 'diagnostic test'/exp OR 'diagnostic equipment'/de OR 'diagnostic kit'/exp) AND ('prediction and forecasting'/exp OR 'predictive validity'/de OR (predict* OR earl* OR subclinic* OR mild):ab,ti) AND ('non invasive procedure'/exp OR (simpl* OR cheap OR 'field level' OR fast OR rapid* OR easy OR ease OR quick*OR novel* OR handheld OR noninvas* OR bedside* OR portable* OR (non NEXT/1 invas*)):ab,ti) NOT ([animals]/lim NOT [humans]/lim)
